# Single-File Water Flux Through Two-Dimensional Nanoporous Membranes

**DOI:** 10.1186/s11671-020-03436-4

**Published:** 2020-11-02

**Authors:** Myung Eun Suk

**Affiliations:** Mechanical Engineering, IT Convergence College of Components and Materials Engineering, Dong-Eui University, Busan, South Korea

**Keywords:** Two-dimensional membrane, Nanopore, Water flux, Membrane hydrophilicity, Molecular dynamics simulation, Diffusion coefficient, Potential of mean force

## Abstract

Recent advances in the development of two-dimensional (2D) materials have facilitated a wide variety of surface chemical characteristics obtained by composing atomic species, pore functionalization, etc. The present study focused on how chemical characteristics such as hydrophilicity affects the water transport rate in hexagonal 2D membranes. The membrane–water interaction strength was tuned to change the hydrophilicity, and the sub-nanometer pore was used to investigate single-file flux, which is known to retain excellent salt rejection. Due to the dewetting behavior of the hydrophobic pore, the water flux was zero or nominal below the threshold interaction strength. Above the threshold interaction strength, water flux decreased with an increase in interaction strength. From the potential of mean force analysis and diffusion coefficient calculations, the proximal region of the pore entrance was found to be the dominant factor degrading water flux at the highly hydrophilic pore. Furthermore, the superiority of 2D membranes over 3D membranes appeared to depend on the interaction strength. The present findings will have implications in the design of 2D membranes to retain a high water filtration rate.

## Introduction

Single-file water transport has been observed in sub-nanometer nanopores involved in synthetic membranes [1, 2] or natural membranes [3, 4]. These single-file water formations in sub-nanometer pores effectively hinder ion translocation by developing a free energy barrier of dehydration [5]. After finding fast water flux rates and high salt rejection rates in carbon nanotube (CNT) membranes [2, 6], many other factors such as rim functionalization, charge assignment, and surface modifications have been studied to understand the transport mechanism and to raise the efficiency of membranes [7–10]. Furthermore, the graphene oxide membranes have been successfully used for ion sieving by adjusting the interlayer spacing of graphene oxide to the sub-nanometer scale [11].

The discovery of two-dimensional (2D) membranes, initiated by graphene [12], has gained significant attention in the field of filtration and desalination membranes [13]. As a result of its one-atom-thick pore width, the frictional pressure loss can be minimized theoretically, and a superior water flux can be obtained [14]. Nanoporous single-layer graphene has been successfully fabricated by using an oxygen plasma etching process, allowing control of pore size [15, 16]. It has been successfully used for desalination membranes by exhibiting nearly 100% salt rejection and high water flux up to 10^6^ g/m^2^ s [16]. High desalination performance is also demonstrated by performing molecular dynamics (MD) simulations [17]. In addition, nanoporous graphene membranes exhibited efficient molecular sieving for gas separation [18, 19] and ion separation [15, 20].

After successful synthesis of graphdiyne [21, 22], other 2D graphene derivatives such as graphyne, graphone, and graphane have attracted great attention as a new class of 2D materials [23, 24]. In addition, surface modifications using pore functionalization or chemical doping have been introduced to extend the functionality of 2D membranes. Nitrogen [25] or nickel [26] doping exhibited superior catalytic activities. Crown-ethers have been embedded in the graphene nanopore for mechanosensitive ion translocation activities [27] or selective ion translocations [20, 28]. Graphene nanopore functionalization using pyridinic nitrogen, fluorine, or hydroxyl has exhibited enhanced desalination efficiency from MD simulations [29–31]. With naturally high porosity, graphyne-3 and graphyne-4 were also proven to be potential candidates for desalination membranes by demonstrating a high water filtration rate and salt rejection rate [32].

Moreover, advanced 2D materials such as silicene [33], germanene [34, 35], hexagonal boron nitride (hBN) [36, 37], and metal organic frameworks (MOF) [38] have been developed and extensively studied in recent years. The development of 2D materials has been extended to structurally asymmetric Janus 2D materials, such as MoSSe [39, 40] and In_2_SSe [41]. New 2D materials such as MOF [42] and MoS_2_ [43] have exhibited an efficient desalination performance using MD simulations. In experiments, MOF membranes as thin as 3 nm have been synthesized and tested for nanofiltration [44]. MoS_2_ as thin as 7 nm has also been synthesized and tested for its desalination efficiency [45]. They both demonstrated high water filtration rates and dye/salt rejection rates. 2D hBN was found to be superior to graphene membranes by exhibiting a higher water permeation rate [46] from an MD study.

Predicting the efficiency of various 2D membranes as water filtration membranes requires understanding the effect of surface chemical properties on the water transport rates. Surface hydrophilicity plays a crucial role in water dynamics at the interface [47]. In the present study, the surface hydrophilicity was tuned by adjusting the membrane–water interaction strength and its effect on the water flow rate was investigated by using MD simulations. To represent the monolayer 2D membranes, a hexagonal graphene structure was selected as the 2D model structure. The single-file water flow through 2D membranes was compared with that through three-dimensional (3D) membranes where water translocation lengths correspond to multiple atomic sizes. To represent the 3D membranes, CNT structure with graphene slabs was used as the 3D model structure.

## Methods

**Fig. 1:**
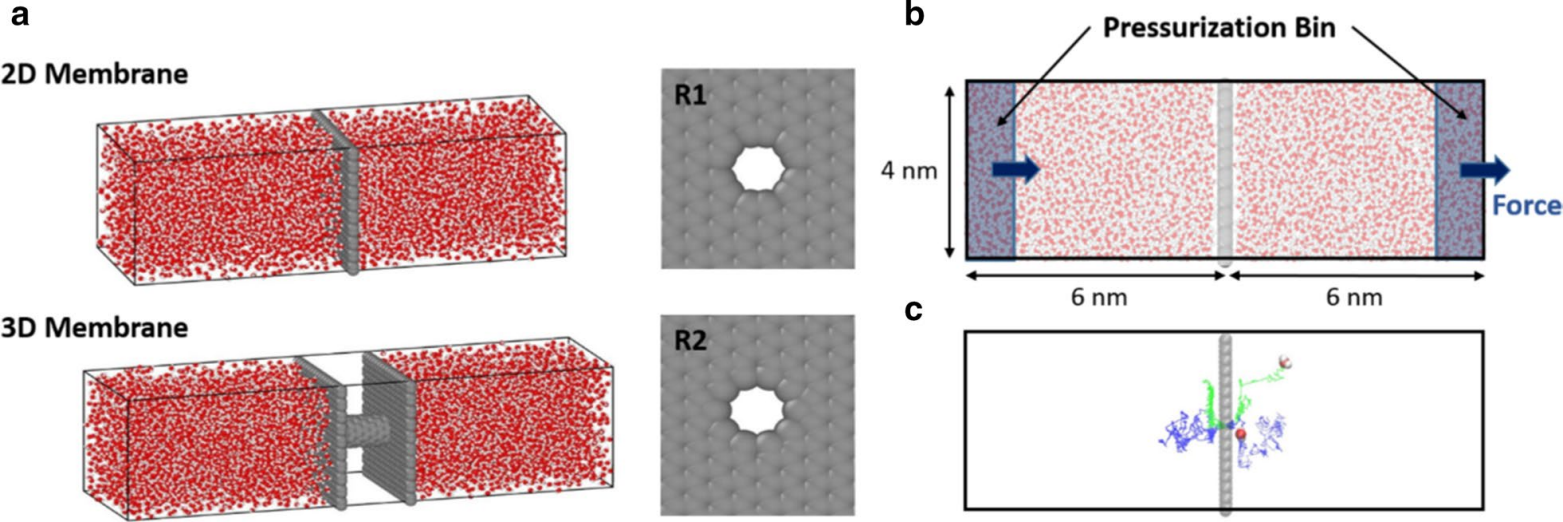
**a** Simulation cells with 2D and 3D membrane structure. R1 shows a pore entrance configuration of the 3D membrane. For the 2D membrane structure, both the R1 and R2 entrance configuration were used. The black box lines represent the periodic boundary of the simulation cells. **b** Application of forces on water molecules during pressure-driven water flow simulations. **c** Representative trajectory paths of water molecules permeating through the 2D membrane

2D membranes and 3D membrane structures were obtained from the geometrical structure of graphene and carbon nanotubes, as shown in Fig. 1. Nanopores in 2D membranes were generated by removing atoms inside the circular region from the pore center (designated as R2). The resulting pore area is hexagonal in shape where the distance between the farthest atoms is approximately 7.52 Å. The 3D membrane structure was obtained by inserting the (6,6) CNT structure between two graphene slabs separated 2.06 nm apart. A slight difference existed between the pore areas of 3D and 2D membranes. An additional 2D membrane structure composed of a CNT rim and graphene slab was generated to eliminate the effect of the pore size difference. The configuration is designated as R1. The pore radius of R1 configuration corresponds to the radius of (6,6) CNT, which is 8.13 Å.

The planar size of the membranes was 4.12 × 4.08 nm. The initial simulation box size was 4.12 × 4.08 × 12 nm for 2D membrane simulations and 4.12 × 4.08 × 14.06 nm for 3D membrane simulations. A periodic boundary condition was applied in the *x*, *y*, and *z* directions along with the simulation box, which is shown in Fig. 1. The membranes were positioned to be perpendicular to the *z*-direction at the center of the simulation box (*z* = 6 nm). The SPC/E water model [48] was used to fill the simulation box as this model is in good agreement with the experimental transport properties such as diffusivity [48, 49] and viscosity [50, 51]. The total number of water molecules was 6474. The non-bonded interaction between water molecules and the membrane was calculated by the Lennard Jones (LJ) interaction,

$${{V}}_{\rm LJ}=4\varepsilon \left[{\left(\frac{\sigma }{r}\right)}^{12}-{\left(\frac{\sigma }{r}\right)}^{6}\right]$$

where $\varepsilon$ is the depth of the potential well, $\sigma$ is the distance between atoms at which the potential is zero, and *r* is the distance between atoms. In these simulations, $\sigma$ is fixed at 0.33 nm, which is the arithmetic mean of the carbon and water distance parameter. The water–membrane interaction strength, $\varepsilon ,$ is changed from 0.026 to 0.415 kcal/mol to tune the hydrophilicity. The interaction strengths used in the present study correspond to 0.25 ${\varepsilon }_{0}$, 0.5 ${\varepsilon }_{0}$, ${\varepsilon }_{0}$, 2 ${\varepsilon }_{0}$, and 4 ${\varepsilon }_{0}$, where ${\varepsilon }_{0}$ is the LJ interaction strength between carbon [52] and oxygen [48].

All simulations were performed using GROMACS software [53]. The time integration was performed using the Leapfrog algorithm with a time step of 1 fs. The Nosè–Hoover thermostat [54] was applied to maintain the temperature at 300 K, with a time constant of 0.1 ps. The cutoff scheme was used in calculating the LJ interaction with the cutoff distance of 12 Å. The long-range electrostatic interactions were calculated by using the particle mesh Ewald (PME) method with a real-space cutoff of 12 Å and reciprocal space gridding of 1.2 Å. During the initial equilibrium simulations, the water pressure normal to the membrane was adjusted to 1 bar by applying the Parrinello–Rahman barostat [55]. After 1 ns of NPT equilibration, the system was further equilibrated using the NVT ensemble for 1 ns. After a total of 2 ns of equilibration, pressure-driven flow was simulated by applying force on the water molecules that reside in the pressurization bin [14, 56]. The pressurization bin of 1 nm length is located at the side of the simulation box, as depicted in Fig. 1b. The external forces acting on water molecules were calculated by $f=\Delta P/NA$, where $\Delta P$ is the desired pressure difference across the membrane, N is the number of water molecules in the pressurization bin, and A is the membrane area. It is known from previous literature that this method is able to maintain the desired pressure drop very well during the course of simulations [14]. The pressure-driven flow was simulated for 10 ns, and the data were collected for 9 ns after 1 ns initialization. During the course of the simulation, the membranes were treated as a rigid material.

After the simulation was performed, the water structure and transport properties were analyzed. The diffusion coefficient in the pore axial direction was calculated by Einstein relations, which is given by

$${D}_{z}=\frac{1}{2}\underset{t\to \infty }{\mathrm{lim}}\frac{\langle {\left|z\left(t\right)-z(0)\right|}^{2}\rangle }{\Delta t}$$

The potential of mean force (PMF) was calculated by integrating forces acting on water molecules through the relations [57],

$$\Delta \mathrm{PMF}=-{\int }_{{z}_{0}}^{z}{\rm d}{z}^{^{\prime}}\langle F({z}^{^{\prime}})\rangle$$

where ${z}_{0}$ is the location of bulk water. ${z}_{0}$ = 3 nm in the present study. In the calculation of ΔPMF and diffusion coefficient profiles in the *z*-direction, cylindrical bins with a radius of 3.8 Å were used along the nanopore axis.

## Results and Discussion

### Water Flux

**Fig. 2:**
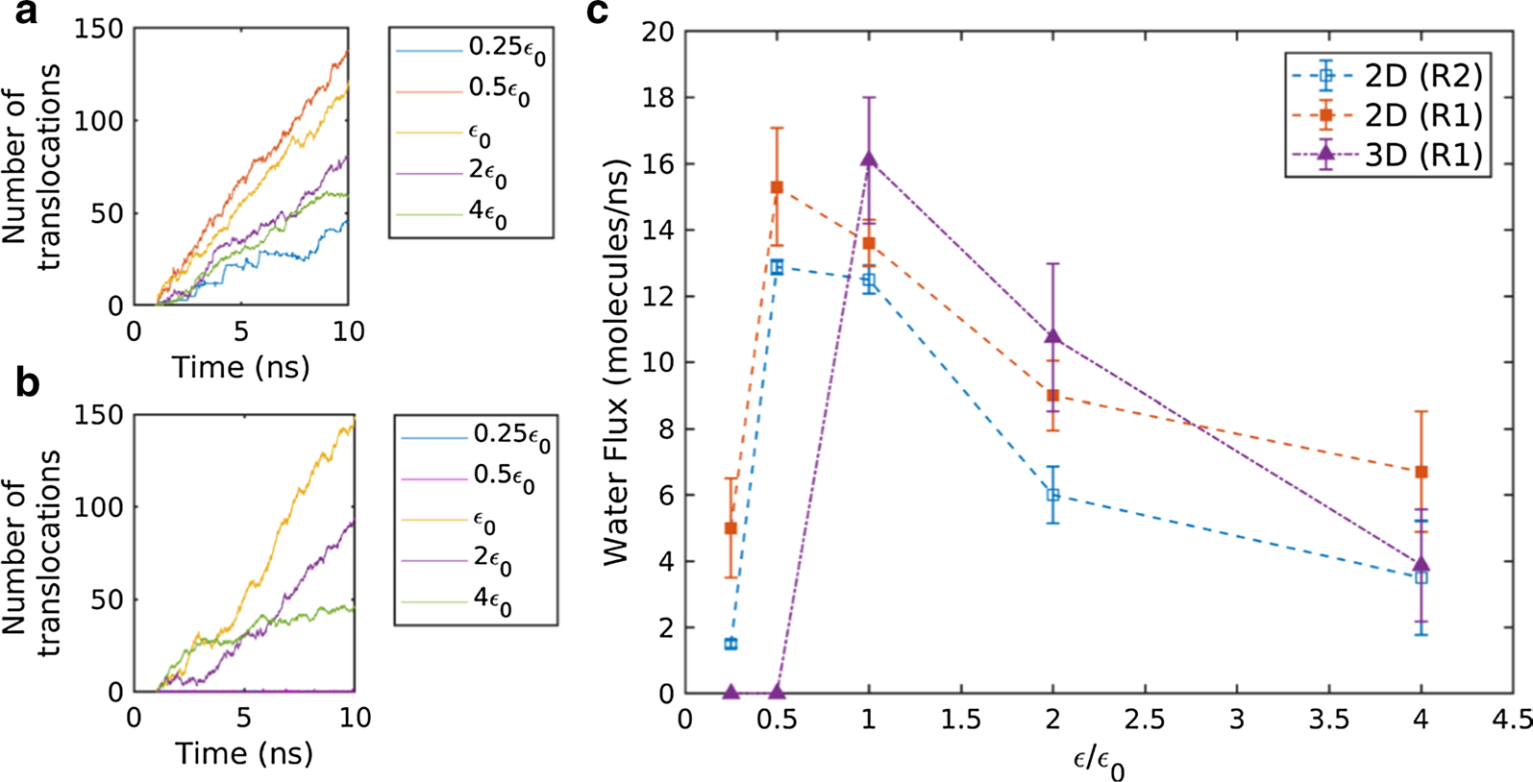
**a** Number of translocated water molecules with time in 2D membranes, **b** number of translocated water molecules with time in 3D membranes, **c** calculated water flux (number of translocated water molecules per ns) variation with the water–membrane interaction strength

During the application of pressure drop across the membrane, the number of water molecules translocating through the membrane was counted, as can be seen in Fig. 2a, b. Figure 2a, b represents the number of water translocations through the 2D (R1) and 3D (R1) membranes, respectively. From the slope of water translocation vs. time, the average water flux was measured. In Fig. 2c, the measured water flux was plotted with the interaction strengths for 2D and 3D membranes. As the interaction strength increases, the water flux sharply increases to a maximum water flux, and then, it monotonically decreases in all membranes. In 2D membranes, the water flux of R1 was slightly higher than that of R2. The difference is a result of the somewhat larger water accessible region of R1.

**Fig. 3:**
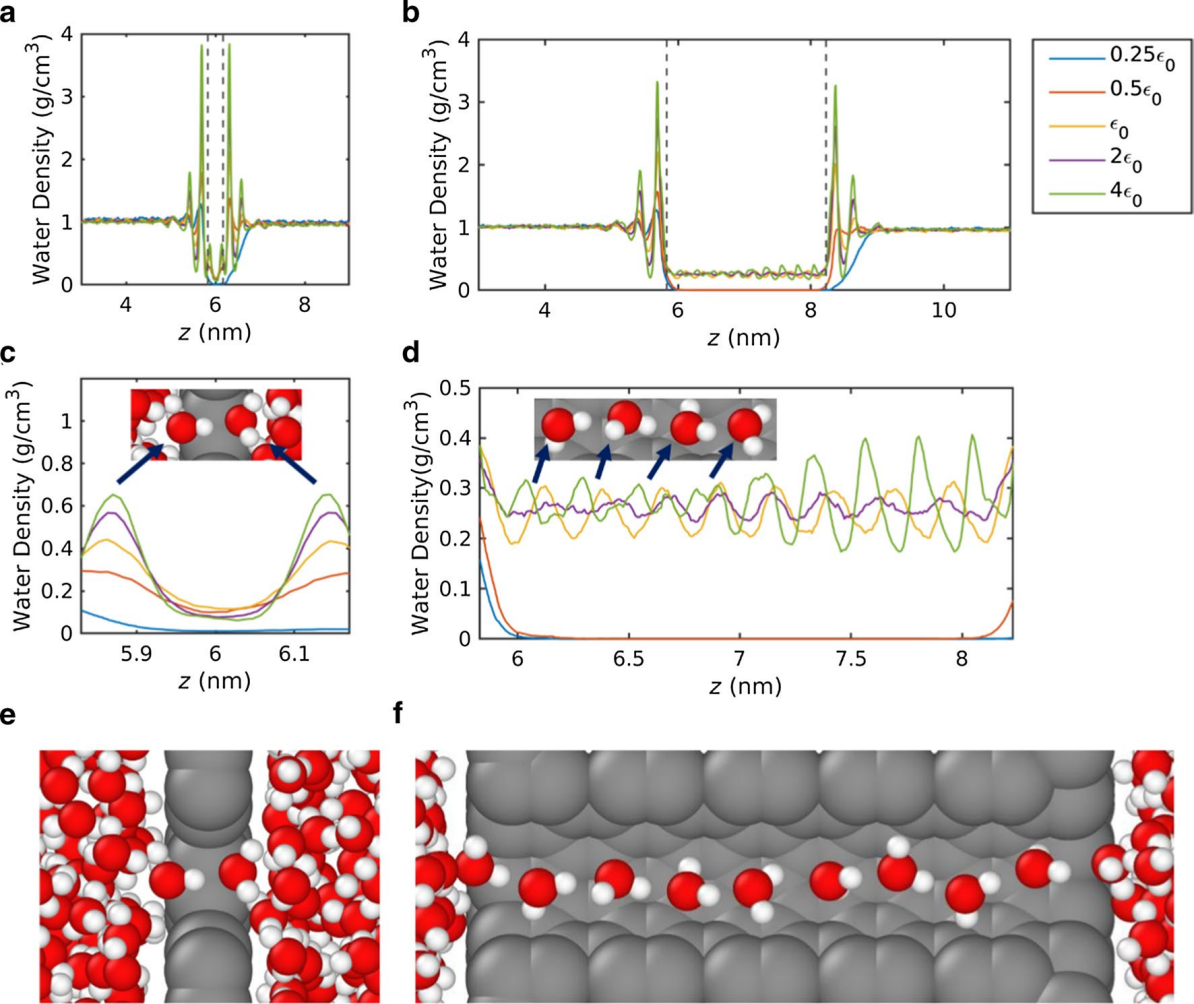
**a**–**d** Water density profile and **e**–**f** visualized single-file water formation during MD. Water density along the axial direction of the nanopore for **a** 2D membranes and **b** 3D membranes. Density was measured in cylindrical bins with a water accessible pore radius. Detailed density profile inside the pore region for the **c** 2D membrane and **d** 3D membrane. Single-file configuration inside the **e** 2D nanopore and **f** 3D nanopore

The minimum water flux to the maximum water flux transition at the low interaction strength owes to the pore dewetting–wetting transition. In nanopores with a sub-nanometer diameter, water molecules are arranged as a single-file chain [1, 58], as can be seen in Fig. 3e, f. The number of hydrogen bonds of water molecules forming a single-file reduces to approximately one and a half [59]. In the formation of the single-file, the lost hydrogen bonding energies are partially compensated by the membrane–water interaction energy [1]. At a low membrane–water interaction strength depicting the hydrophobic pore, the membrane–water interaction does not provide enough compensation to form the single-file chain. Such dewetting behavior is confirmed in both the pressure-driven and equilibrium simulations, by plotting the density profile and measuring occupation number (see details in "Water Density" section and "Water Occupation in Nanopores" section).

The 2D and 3D membranes exhibited differences in the threshold interaction strength. The threshold interaction strength of the 3D membranes was higher than that of the 2D membranes. Inside the sub-nanometer pore, a partial chain or individual water molecules are energetically unfavorable. Therefore, complete chain formation inside the pore is a prerequisite in the wetting of the sub-nanometer pore. A relatively short chain length and closely located bulk water baths enable the wetting of 2D membranes at a relatively low interaction strength. Due to such a difference in threshold interaction strength, the water flux of 2D membranes was higher than that of the 3D membrane at low interaction strengths (0.25 ${\varepsilon }_{0}$ and 0.5 ${\varepsilon }_{0}$).

On the threshold interaction strength wetting nanopores, the maximum water flux is reached. Then, water flux decreases with increase in interaction strength. It has been reported that hydrophobic surfaces promote the boundary slip, and subsequently enhance water flux [60–62]. The continuum hydrodynamics also govern the enhanced water flux when the slip boundary condition is applied. The validity of the same mechanism on a single-file flux and the 2D membrane is unclear because of sub-nanometer dimensions in the pore axial and radial direction. To explain the water flux decreasing with increasing hydrophilicity, water dynamics and energetics were investigated (see "Water Diffusivity" and "Potential of Mean Force" sections). Note that the decrease in water flux was more significant for 3D membranes compared with 2D membranes. At moderate interaction strength (${\varepsilon }_{0}, 2{\varepsilon }_{0}$), 3D membranes are superior to 2D membranes, while the reverse is true at high interaction strength (4 ${\varepsilon }_{0})$.

### Water Density

Water density profiles along the pore axial direction are plotted in Fig. 3a–d. Water density is measured using the cylindrical bins with the pore radius to access the density profile in the open pore region. Figure 3a and b represents the water density profile with 2D and 3D membranes, respectively, with the pore region indicated by the dashed lines. The width of the pore region is defined as the van der Waals diameter of membrane atoms. As the center of membrane atoms is located at *z* = 6 nm, pore regions are defined as *z* = 5.83–6.17 nm for 2D membranes, and *z* = 5.83–8.23 nm for 3D membranes. In Fig. 3c, d, the water density inside the pore region is displayed.

In the proximal region of the pore entrance, significant density peaks and valleys, representing a layered water structure, are clearly observed. The layered water structure near the solid walls has been reported by previous MD [63] and experimental studies [64]. As the pore radius is smaller than the distance within which van der Waals interactions act (~ 1.2 nm), the layered water structure did not vanish despite the pore being open. It is observed from the density oscillations that the magnitude of the density peak increases with increase in interaction strength.

The density peaks inside the pore region indicate the favorable sites of water molecules forming the single file. In 2D nanopores, two density peaks indicate that two water molecules form a stable single file. In 3D nanopores, eight to nine density peaks were observed, indicating that a longer water chain was built (Fig. 3e, f). The zero water density inside the pore region indicates that no water molecules permeate through the membranes. In 2D nanopores, the water density is close to zero with an interaction strength of 0.25 ${\varepsilon }_{0}$; therefore, the water flux was nominal for 2D nanopores with an interaction strength of 0.25 ${\varepsilon }_{0}$. In 3D nanopores, water density is zero for interaction strengths of 0.25 ${\varepsilon }_{0}$ and 0.5 ${\varepsilon }_{0}$, meaning the water flux was measured as zero for 3D nanopores with those interaction strengths.

### Water Occupation in Nanopores

**Fig. 4:**
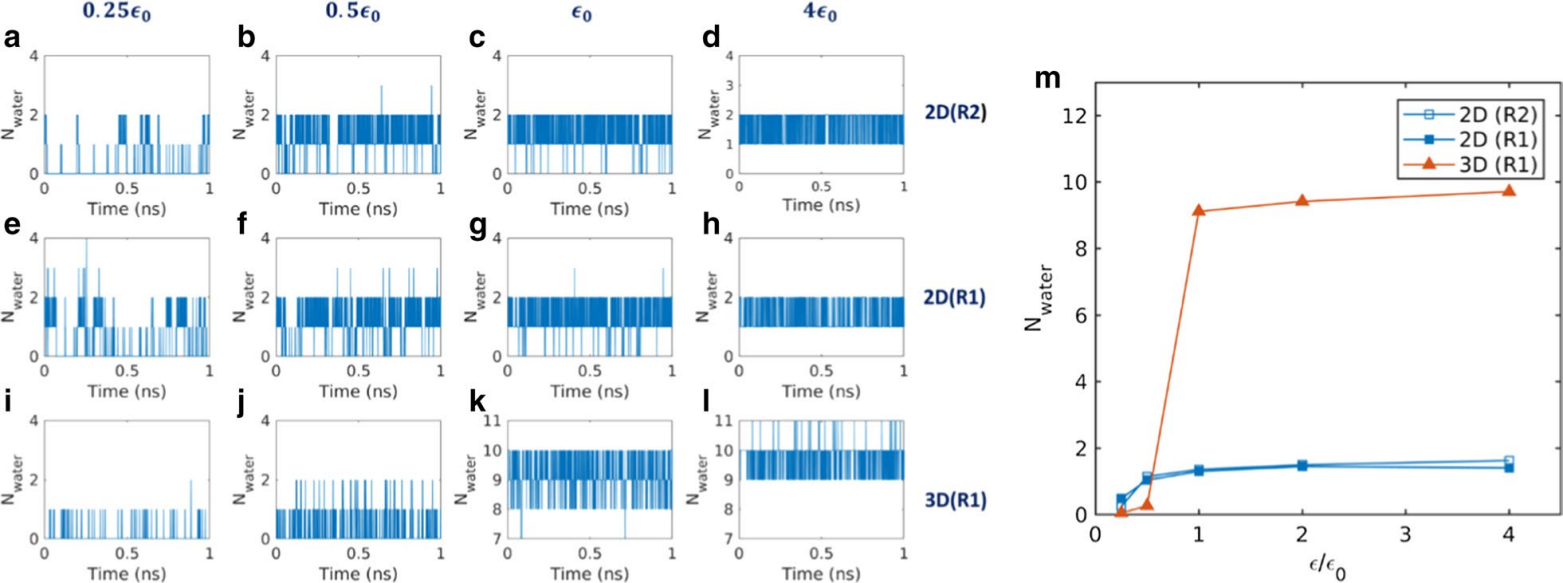
Water occupation number inside the nanopore for **a**–**h** 2D and **i**–**l** 3D membranes. The membrane–water interaction strengths are 0.25 ${\epsilon }_{0}$ for **a**, **e**, and **i**, 0.5 ${\epsilon }_{0}$ for **b**, **f**, and **j**, ${\epsilon }_{0}$ for **c**, **g**, and **k**, and 4 ${\epsilon }_{0}$ for **d**, **h**, and **l**. Average occupation numbers vary with the interaction strength (**m**)

The equilibrium water dynamics were also investigated by running equilibrium simulations without the external pressure difference. The water occupation numbers of nanopores were measured by counting the instantaneous amount of water molecules inside the pore region during each time frame. Figure 4a–l displays the occupation number with time for the various interaction strengths of the 2D and 3D membranes. As can be seen in Fig. 4, water occupation demonstrates the water empty-filling two-state transition of the nanopores. This is known to be characteristic of single-file water, as the partially filled (broken single-file) state is energetically unfavorable [1]. At a low interaction strength of 0.25 ${\epsilon }_{0}$, the empty state (0–1 water occupancy) is more populated for both 2D and 3D membranes. At this strength, the average occupation number was 0.37 for 2D membranes and 0.05 for 3D membranes. At an interaction strength of 0.5 ${\epsilon }_{0}$, the filled state (1–2 water occupancy) is more populated for 2D membranes, while the empty state is still populated for 3D membranes. At this strength, the average water occupation number was 1.1 for 2D membranes and 0.3 for 3D membranes. At an interaction strength of ${\epsilon }_{0}$, the occupation number is populated with 8–10 for 3D membranes. This indicates that 3D membranes are in the filled state with an interaction strength of ${\epsilon }_{0}$.

The variation of average water occupation number with the interaction strength is displayed in Fig. 4m. The empty-filling two-state transitional behavior was also observed with the interaction strength. A sharp transition is clearly observed for 3D membranes as the occupation number jumps from a nominal number to a high number, and then slightly increases with increase in interaction strength. Similar transitional behavior is observed in 2D membranes; however, 2D membranes exhibit a moderate transition owing to the short single-file chain length and closely located bulk water bath, which govern a relatively favorable transitional state.

The transitional behavior of empty-filling (dewetting–wetting) states supports the water flux variation at a low interaction strength. Below the threshold interaction strength, the water flux due to the applied pressure drop was zero or nominal. At an interaction strength of 0.5 ${\epsilon }_{0}$, the water flux for the 2D membrane was much higher compared with the 3D membrane. At this interaction strength, the 2D membrane is in the wetting state, while the 3D membrane is in the dewetting state. Therefore, it can be concluded that water dewetting is responsible for water flux variations at a low interaction strength. Unfortunately, the water occupancy cannot explain the water flux decreases with a higher interaction strength.

### Water Diffusivity

**Fig. 5:**
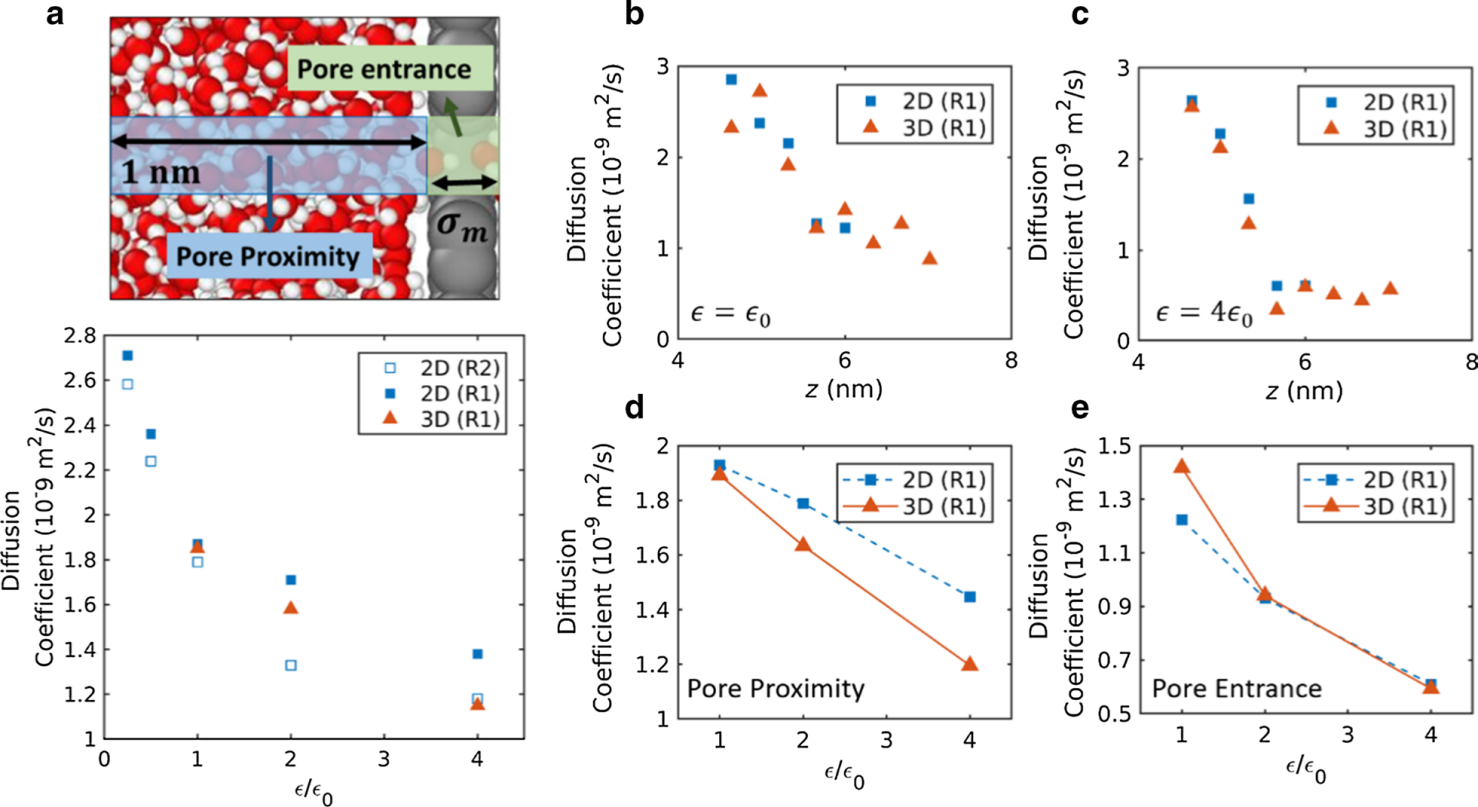
Water diffusion coefficients in pore proximity and pore entrance region. The pore proximity region is defined as the cylindrical region with a pore radius and 1 nm of length. The pore entrance region is defined as the cylindrical region with a pore radius and van der Waals diameter. **a** Diffusion coefficient variations with the interaction strength. Diffusion coefficients are measured in areas, including both pore entrance and proximity region. **b**, **c** Diffusion coefficient profile along the pore axial direction for an interaction strength of **b** ${\epsilon }_{0}$ and **c** 4 ${\epsilon }_{0}$. **d**, **e** Diffusion coefficient variation with interaction strength in the **d** pore proximity region and **e** pore entrance region

To further investigate water dynamics, water diffusion coefficients were calculated from equilibrium simulations. In density profiles, large oscillations at the proximal pore region were observed, which indicates a layered water structure. The amplitude of density oscillation increased with increase in interaction strength. In order to take account of such structural effects, water diffusion coefficients in the pore proximity and entrance regions were calculated and plotted in Fig. 5a–e. Figure 5a exhibits diffusion coefficients of water molecules in various areas, including both proximity and entrance regions. It is clear that diffusion coefficients decrease with increase in interaction strengths. Therefore, it can be concluded that the decrease in water diffusivities contributed to the water flux decrease with increase in interaction strength above the threshold interaction strength.

The profiles of diffusion coefficients in the pore axial direction are shown for a moderate interaction strength (${\epsilon }_{0}$) and high interaction strength (4 ${\epsilon }_{0}$) in Fig. 5b, c, respectively. In Fig. 5b–e, the same pore configuration (R1) for 2D and 3D nanopores is compared to eliminate any effect caused by the pore configuration difference. It was observed that the diffusion coefficients gradually decreased from the bulk diffusivity (~ 2.7 × 10^–9^ m^2^/s [49]) as they approached the pore entrance. The decrease in the diffusion coefficient may be the consequence of a combination of the pore confinement effect and the water layering effect. Membrane hydrophilicity is likely to reduce the diffusion coefficient by two different mechanisms, that is, inducing a highly layered structure in pore proximity and increasing frictional force in the pore entrance region. In separate diffusion coefficient calculations in pore proximity and entrance regions (see Fig. 5d, e), the diffusion coefficients decreased with increase in interaction strength in both areas.

The diffusion coefficients for 3D membranes were slightly higher or comparable to that of 2D membranes in the pore entrance regions. In contrast, the diffusion coefficients for 3D membranes were smaller than that of 2D membranes in the pore proximity, and the difference is significant at the high interaction strength (4 ${\epsilon }_{0}$). In the pressure-driven flow simulation, the water flux through 3D membranes exhibited a more significantly decreased rate with the interaction strength compared to 2D membranes. This resulted in comparable or higher water flux for 3D membranes at a moderate interaction strength (${\epsilon }_{0},$ 2 ${\epsilon }_{0}$), and higher water flux for 2D membranes at a high interaction strength (4 ${\epsilon }_{0}$). The diffusivities in pore proximity appear to be the main cause of such reversed water flux at a high interaction strength.

### Potential of Mean Force

**Fig. 6:**
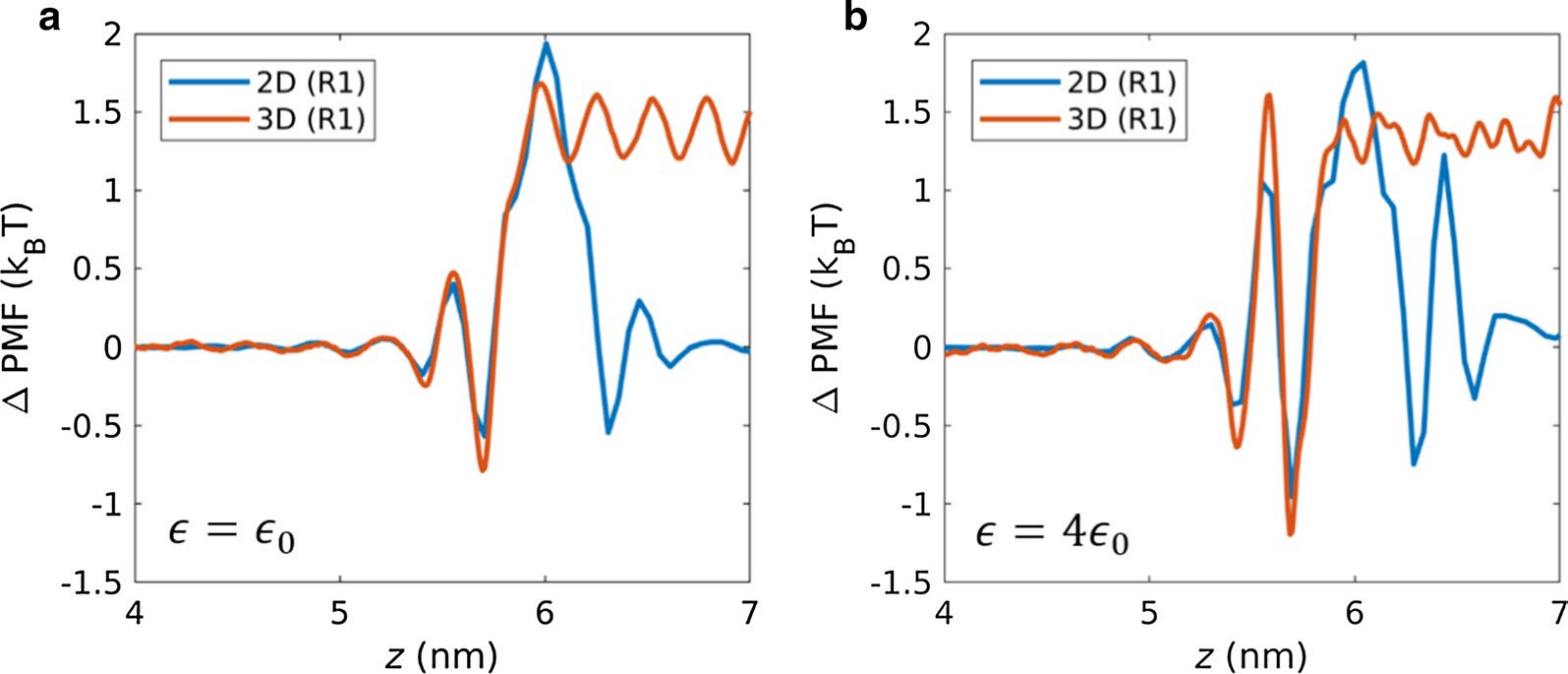
PMF profiles along the pore axial direction for a **a** moderate interaction strength (${\epsilon }_{0})$ and **b** high interaction strength ($4{\epsilon }_{0})$

To further investigate the superiority of the membranes, which depends on the interaction strength, the 2D and 3D PMF profiles were compared for the moderate interaction strength (${\epsilon }_{0})$ and high interaction strength (4 ${\epsilon }_{0}$). The PMF profiles of 2D and 3D nanopores are compared in Fig. 6. The PMF profile shows local maxima, representing the free energy barrier that water molecules should overcome in order to transport through the membranes. From the PMF profiles, two major PMF energy barriers were identified at the pore entrance region (*z* = 6 nm) and the pore proximity region (*z* $\approx$ 5.5 nm). At an interaction strength of ${\epsilon }_{0}$, the proximity energy barrier did not exhibit a significant difference between 2 and 3D. At a high interaction strength of 4 ${\epsilon }_{0}$, the energy barriers at the proximity were both increased, but with a higher magnitude for 3D membranes compared with the 2D membranes. This confirms that the pore proximity is the main factor for reversed water flux at a high interaction strength.

With increase in interaction strength (${\epsilon }_{0}$ → $4{\epsilon }_{0}$), the pore entrance energy barrier changed from 1.94 to 1.82 for 2D membranes, and 1.68 to 1.45 for 3D membranes. There is a slight decrease in the entrance energy barrier with increasing membrane–water interaction energy. On the other hand, with increase in interaction strength (${\epsilon }_{0}$ → $4{\epsilon }_{0}$), the proximity energy barrier changes from 0.4 to 1.05 for 2D membranes, and 0.47 to 1.61 for 3D membranes. From the energetic viewpoints, a water flux decrease with increase in interaction strength is predominantly due to the energy barrier increasing in the proximal pore region. It is also related to the higher reduction of water flux for 3D membranes, compared with 2D membranes. The total energy barrier for 2D membranes (2.34 k_B_T) is slightly higher than that of 3D (2.15 k_B_T) membranes when the membrane–water interaction is moderate (${\epsilon }_{0}$). Due to the significant increase in proximity energy barrier for 3D membranes, their total energy barrier (3.06 k_B_T) is higher than that of 2D membranes (2.87 k_B_T) in the case of a high interaction strength (4 ${\epsilon }_{0})$. Therefore, ΔPMF quantitatively supports the superiority of 2D membranes at a high interaction strength (4 ${\epsilon }_{0})$ and 3D membranes at a moderate interaction strength (${\epsilon }_{0})$.

For non-single-file water flow through larger pore sizes, it is presumed that 2D membranes are dominant over 3D membranes regardless of interaction strength. The wetting–dewetting behavior with the interaction strength was observed for CNT membranes with larger pore sizes from previous literature [65]. The threshold interaction strength decreased with increasing pore sizes [65]. Due to the closely located water reservoirs and short pore length, the 2D membranes will exhibit lower threshold interaction strength compared to the 3D membranes, which is consistent with the results for single-file flow. Thus, 2D membranes are likely to show higher water flux through larger pore sizes compared to that through 3D membranes when interaction strength is low. For interaction strength above the threshold, water flux through 2D membranes may still be higher than that of 3D membranes as opposed to the single-file water flow. The PMF energy barrier at the pore proximity will not affect the water flow as much, and frictions between the membrane wall and water molecule will become a dominant factor affecting the water flux. Previous literature has reported that water flux through CNT membranes increases with decrease in CNT length for non-single-file flow [66, 67]. Additionally, for non-single-file flow, a higher water flux through graphene membranes was observed compared to that through CNT membranes [14].

## Conclusions

In the present study, the effect of the membrane–water interaction strength on the single-file water flux was investigated. Due to the recent advances in two-dimensional membranes, hexagonal 2D membrane structures were considered and compared with the 3D tube type structure. The main observations are as follows: (1) water flux is zero or nominal below the threshold interaction strength, (2) the threshold interaction strength is lower for 2D membranes compared with 3D membranes, (3) water flux decreases with increase in interaction strength when the interaction strength is larger than the threshold interaction strength, and (4) the decrease in water flux was more significant for 3D membranes compared with 2D membranes.

The zero or nominal flux at a low interaction strength was due to the dewetting behavior, which was supported by the small occupation number and water density inside the pore. Above the threshold interaction strength wetting the pore, the water flux decreases with increase in interaction strength. The increase in the interaction strength resulted in an increased PMF energy barrier and decreased diffusion coefficients at the pore proximity, consequently reducing the water flux. In addition, the water structure and dynamics in the pore proximity were more affected by the interaction strength in the 3D membrane compared with that of the 2D membrane. It resulted in the higher reduction of water flux for 3D membranes, compared with the 2D membranes.

Due to the complicated single-file flux dependency on the interaction strength and membrane dimensions, the superiority of 2D membranes over 3D membranes appears to depend on the interaction strength. For a moderate interaction strength (l ${\epsilon }_{0}$,${2\epsilon }_{0})$, the 3D membrane shows a slightly higher water flux compared with the 2D membranes. For a low (0.5${\epsilon }_{0}$) and high interaction strength (4${\epsilon }_{0}$), the 2D membrane shows a higher water flux than the 3D membranes. To conclude, the superiority of 2D membranes over 3D membranes depends on the membrane hydrophilicity due to the wetting–dewetting transition and diffusion dynamics in pore proximity. The present findings will be useful in the design and manipulation of 2D membranes to retain a high filtration flux.

## Data Availability

The datasets supporting the conclusions of this article are included within the article, and further information about the data is available from the corresponding author on reasonable request.

## Abbreviations

2D: Two dimensional
3D: Three dimensional
CNT: Carbon nanotube
MD: Molecular dynamics
hBN: Hexagonal boron nitride
MOF: Metal organic framework
LJ: Lennard–Jones
PMF: Potential of mean force
